# Diabetes and Tooth Loss in a National Sample of Dentate Adults Reporting Annual Dental Visits

**Published:** 2007-06-15

**Authors:** Julie M Kapp, Suzanne Austin Boren, Shumei Yun, Joseph LeMaster

**Affiliations:** University of Missouri–Columbia, Columbia; Department of Health Management and Informatics, School of Medicine, University of Missouri–Columbia, and Health Services Research and Development Program, Harry S. Truman Memorial Veterans’ Hospital, Columbia, Mo; Department of Health and Senior Services, Missouri Department of Health, Jefferson City, Mo; Department of Family and Community Medicine, School of Medicine, University of Missouri–Columbia, Columbia, Mo

## Abstract

**Introduction:**

Periodontal disease has been associated with tooth loss and reported as more prevalent among people with diabetes than among those without diabetes. Having an annual dental examination is a national goal of *Healthy People 2010*. Our objective was to examine whether an association exists between diabetes and tooth loss among a population reporting an annual dental visit.

**Methods:**

We used data from the 2004 Behavioral Risk Factor Surveillance System to examine the association between self-reported diabetes and tooth removal due to decay or periodontal disease among 155,280 respondents reporting a dental visit within the past year. We calculated prevalence estimates, odds ratios, and 95% confidence intervals. Multiple logistic regression allowed for adjustment.

**Results:**

The overall prevalence of tooth removal among the people in the study was 38.3%. People with diabetes had a significantly higher prevalence of tooth removal. In a multivariable model adjusting for selected covariates, respondents with diabetes were 1.46 times as likely (95% CI, 1.30–1.64) to have at least one tooth removed than respondents without diabetes. A stronger association between diabetes and tooth loss was observed among people in the younger age groups than among those in the older age groups.

**Conclusion:**

Even among people reporting a recent dental visit, diabetes was independently associated with tooth loss. Multidisciplinary efforts are needed to raise awareness of the risk of tooth loss among younger people with diabetes. Good oral hygiene as well as annual dental examinations are important for preventing tooth loss.

## Introduction

Diabetes mellitus is a major health problem in the United States, where approximately 20.8 million people, or 7% of the population, have the disease (1). The Behavioral Risk Factor Surveillance System (BRFSS), a national state-based, random-digit–dialed telephone survey of the noninstitutionalized U.S. population aged 18 years or older, conducted annually by the Centers for Disease Control and Prevention (CDC), provides population-based data on diabetes (2). CDC examined BRFSS data collected during 2002 through 2004 and found that only four in 10 adults with diabetes received all three recommended preventive care services (an annual foot examination, an eye examination, and a biannual glycosylated hemoglobin [HbA1c] test) (3). However, that study did not assess whether the participants reported an annual dental visit. A limited number of population-based studies have examined the association between diabetes and tooth loss (4).

Almost one third of people with diabetes are not aware that they have the disease (1), and many do not receive a diagnosis until after complications develop, which include periodontal disease (5). Periodontal disease, including gingivitis, and dental caries are prevalent in the U.S. population; for example, the prevalence of dental caries is 84.7% for adults aged 18 or older (6). Periodontal disease and dental caries account for most tooth loss, and their impact increases with age (6-8). Periodontal disease is more prevalent and more severe among people with diabetes than among people without diabetes (9,10); studies indicate that periodontal destruction can occur even among children and adolescents (11). The increased risk of periodontal disease among people with diabetes is not surprising, given that diabetes is associated with impaired wound healing (12), exaggerated monocyte response to dental plaque antigens (12), and salivary pH and buffering capacities (13). However, the association between diabetes and oral health is yet to be fully explained. Studies report that causal relationships between diabetes and oral health are not yet established (12), are bidirectional (14), or that no significant differences exist among data on saliva and caries between people with insulin-dependent diabetes mellitus and people without when the disease is well controlled (15).

*Healthy People 2010* set a national goal that 75% of people with diabetes have an annual dental examination (objective 5–15) (16). However, a study of BRFSS respondents found that age-adjusted estimates of dental visits among dentate adults with diabetes exceeded 71% in only seven states (9). A case-control study of adults with type 1 diabetes found that most people with diabetes were unaware of the oral health complications of the disease (17). The age-adjusted rate for dentate people with diabetes who visited a dentist within the preceding 12 months was 66%, whereas the age-adjusted rate for people without diabetes was 73%; the leading reason for not visiting a dentist was perceived lack of need (18).

However, the percentage of those who reported having an annual dental visit (65%) is comparable with the percentage who reported a foot examination (68%) or a dilated eye examination (62%) (18). The comparable rates suggest multiple levels of self-care, and the association between diabetes and tooth loss might be attenuated among people who actively seek dental care. This study is the first to investigate whether an independent association exists between diabetes and tooth loss among a subset of BRFSS respondents who indicated access to dental services. We used a sample of dentate respondents from the 2004 BRFSS who reported a dental visit in the past year.

## Methods

We analyzed data from the 2004 BRFSS (excluding data from U.S. territories). The BRFSS is a cross-sectional design using complex survey sampling, which considers the number of adults and telephones in the household and telephone coverage to account for differences in the probability of selection (2). The data contain no identifying information and are free to the public (19,20). Our study was approved by the institutional review board at the University of Missouri–Columbia.

The study sample consisted of people who responded to the question, "Have you ever been told by a doctor that you have diabetes?" We compared those who responded yes with those who responded no. In 2004, the BRFSS also included the following response options: "Yes, but told only during pregnancy" and "No, told only prediabetes or borderline diabetes." People selecting the latter two options were excluded from the study because of the small sample size.

Tooth loss was our outcome of interest. The survey asked, "How many of your permanent teeth have been removed because of tooth decay or gum disease? Do not include teeth lost for other reasons, such as injury or orthodontics." Because of differences in oral health care use and perspectives between dentate and edentate populations (21), we restricted our sample to dentate individuals who responded "None," "One to five," or "Six or more but not all."

We further restricted the sample in two ways. The BRFSS asks, "How long has it been since you last visited the dentist or a dental clinic for any reason?" We included only those who responded "Within the past year." BRFSS also asks, "Are you limited in any way in any activities because of physical, mental, or emotional problems?" We included only those who responded negatively, because a lack of such problems might facilitate access to care and estimate a less severe level of diabetes. We did not restrict our sample to older adults because of the high prevalence of diabetes among people in younger age groups. The age distribution of any tooth removal due to decay or gum disease in this sample ranged from ages 18 to 99.

The following covariates of interest were selected a priori on the basis of theoretical relevance and were grouped as demographics or access to health care. Demographics included age groups (18 to 44, 45 to 64, and ≥65), sex, marital status (married versus divorced, widowed, separated, never married, or member of an unmarried couple), race (African American or black, white, or other race), annual household income ($0 to <$15,000; $15,000 to <$25,000; $25,000 to <$35,000; or ≥$35,000), education (less than high school graduate, high school graduate, some college, or college graduate), and if currently employed or self-employed (versus out of work, homemaker, student, retired, or unable to work). Recognizing that smoking is a strong risk factor for periodontal disease and tooth loss and is also a proxy for self-care, we included a question on having ever smoked ("Have you ever smoked at least 100 cigarettes in your entire life?"). Questions on access to health care included questions on cost barriers ("Was there a time in the last 12 months when you needed to see a doctor but could not because of cost?"), ability to identify at least one primary health care provider ("Do you have one person you think of as your personal doctor or health care provider?"), and health care coverage ("Do you have any kind of health care coverage, including health insurance, a prepaid plan such as a health maintenance organization or a government plan such as Medicare?").

### Statistical analyses

We calculated prevalence estimates and 95% confidence intervals (CIs). We included diabetes and covariates in the multivariable logistic regression model to estimate prevalence odds ratios (ORs) of tooth loss. We used a two-tailed test on the interaction between diabetes status and age group and reported stratified ORs when an interaction was present. A multinomial logistic regression model examined level of tooth removal. We used the C statistic, or area under the receiver operating characteristic (ROC) curve (22), to estimate model discrimination. We used SAS Version 9.0 (SAS Institute Inc, Cary, NC) to accommodate the complex survey sampling design.

## Results

The final 2004 BRFSS sample of adults who reported a dental visit within the past year included 155,280 unweighted observations. Table 1 presents the characteristics of the sample; 4.6% of the sample reported having diabetes, 38.3% had at least one tooth removed, and 61.8% had no tooth loss. More than half (55.7%) were aged 18 to 44, and 82.1% were white. Most (68.6%) had an annual household income of $35,000 or more, and 38.8% were college graduates. Most (68.9%) were employed, were married (63.1%), did not report a cost barrier to health care access (91.9%), had at least one primary health care provider (83.7%), had health care coverage (89.2%), and had not smoked 100 or more cigarettes (60.4%).

Table 1 also presents prevalence estimates by levels of tooth loss. Respondents with diabetes indicated a significantly higher prevalence of losing both one to five and six or more teeth, compared with no tooth loss. Other variables that indicated significantly higher prevalence of tooth loss compared with no tooth loss included the following: age starting at 45, black race, income less than $35,000, high school graduate or less than a high school graduate, not employed or self-employed, cost barrier to health care, can identify at least one primary health care provider, and smoked at least 100 cigarettes in lifetime.

In a crude logistic regression model, respondents reporting diabetes were significantly more likely to have had at least one tooth removed than respondents not reporting diabetes (prevalence OR, 2.72; 95% CI, 2.48–2.98). In a multivariable model adjusting for covariates from Table 1, respondents with diabetes were 1.46 times as likely (95% CI, 1.30–1.64) to have had at least one tooth removed than respondents without diabetes (C statistic = 0.75) (results not shown in Tables).

Table 2 presents the results of a crude and multivariable multinomial logistic regression analysis for diabetes status by level of tooth removal among this sample of respondents who reported a dental visit within the past year and no activity limitation. Unadjusted results indicate that reporting diabetes is associated with an increased likelihood of tooth removal compared with no tooth removal, with a prevalence OR of 1.64 (95% CI, 1.49–1.80) for one to five teeth removed and a prevalence OR of 3.16 (95% CI, 2.82–3.55) for six or more (but not all) teeth removed. The results were attenuated in a fully adjusted model, with a prevalence OR of 1.11 (95% CI, 0.99–1.24) for one to five teeth removed, and a prevalence OR of 1.46 (95% CI, 1.27–1.68) for six or more (but not all) teeth removed compared with no teeth removed. A significant interaction was found between diabetes status and age group (*P* = .004), and the results of a stratified model are presented in Table 2 and the Figure. Findings stratified by age group were significant for respondents aged 18 to 44, mixed for respondents aged 45 to 64, and not significant for respondents aged 65 or older. Model discrimination was good, increasing with level of tooth removal, as indicated by the C statistic.

**Figure:**
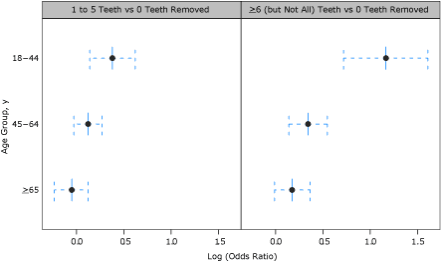
Stratified analysis of the association between diabetes and levels of tooth removal due to decay or gum disease. The model was adjusted for race, sex, income, education, employment, marital status, cost barriers, ability to identify primary health care provider, health care coverage, and having smoked at least 100 cigarettes in lifetime. Source: 2004 Behavioral Risk Factor Surveillance System (20).

**Table:** 

## Discussion

This study examines the association between tooth loss and diabetes among a national, population-based sample of adults, using reported dental visit within the past year as a proxy for access to dental care. *Healthy People 2010* (Part A, Chapter 6) suggests that people with disabilities may have more comorbidities and barriers to health services or medical care (16). Additionally, the rate of amputations among people with diabetes is 10 times higher than among people without diabetes (5). By restricting our sample to people who reported no limitations in their activities due to physical, mental, or emotional problems, we attempted to approximate a sample with less debilitating diabetes and fewer barriers to health care access, therefore attaining more conservative results. In a post hoc analysis that did not restrict the sample by activity limitation due to physical, mental, or emotional problems, results of the final multinomial model did not change by more than 10% (data not shown).

The results of our study contribute to gaps in the existing literature by showing that a significant association exists between diabetes and tooth loss, independent of whether respondents had received dental care within the past year. Findings suggest a dose-response across age groups, with the strength of association progressively weaker in older age groups. The stronger association among younger age groups may suggest more severe periodontal disease resulting from worse dental self-care among younger respondents, more severe diabetes among younger age groups, or competing comorbidities among those aged 65 or older contributing to early mortality. With the latter interpretation, compromised immune response among people with diabetes may increase susceptibility toward oral disease, and oral disease is associated with increased risk for cardiovascular disease and stroke, among other diseases (12).

Okoro et al report a relationship between self-reported tooth loss and heart disease (23) using the BRFSS questions, "Have you ever been told by a doctor, nurse, or other health professional that you have high blood pressure [hypertension]?" and "Have you ever been told by a doctor, nurse, or other health professional that your blood cholesterol is high [hypercholesterolemia]?" Because these questions were not asked in every state in 2004, we excluded them a priori from our main analysis of the association between diabetes and tooth loss. Post hoc adjustments for these variables in the multivariable model of any versus no tooth loss indicate that diabetes remains significantly associated with tooth loss, with an adjusted OR of 1.58 (95% CI, 1.17–2.14) and a C statistic of 0.75.

Our study has several limitations. BRFSS data are self-reported and therefore subject to reporting and recall bias. Some important factors were unknown, including the routine oral preventive care (e.g., tooth brushing, flossing) of the respondents. Although a dental visit within the past year was used as a proxy for access to dental care, the purpose of the reported dental visit was not known and could be attributed either to preventive care, tooth removal, or both. Finally, because the BRFSS data are cross sectional, we do not know when respondents lost their teeth in relation to their diabetes status, and we cannot suggest causality. We are limited to the observation that there appears to be a relationship between diabetes and tooth loss due to decay or gum disease among respondents reporting a dental visit within the past year.

We found an especially strong association between diabetes and tooth loss among younger respondents. These findings need to be confirmed by studies that control for temporality and frequency of good oral hygiene. No prior studies investigating tooth loss reported increasing age-related risk among younger age groups. These findings have even stronger implications because of the epidemic of overweight and obesity among children and adults (24,25), and the suggested increase in type 2 diabetes among children (1). These findings need further investigation. The American Diabetes Association's Standards of Medical Care in Diabetes do not include specific recommendations for oral health (26), perhaps because of a fragmented health care system (26) with little interaction between the dental and medical professions (27). Eighty-four percent of our respondents indicated they have at least one person they think of as their personal doctor, suggesting that mechanisms are in place and perhaps underused for primary care physicians to advise patients — especially patients with diabetes — to visit their dentist. It is important to make people with diabetes aware of their high risk for tooth loss and underscore the importance of good oral health and preventive care beyond annual dental examinations, which may also help in glycemic control.

## Figures and Tables

**Table 1:** Characteristics of U.S. Adults (N = 155,280) Who Reported a Dental Visit in the Past Year and No Activity Limitation, By Number of Teeth Removed, 2004 Behavioral Risk Factor Surveillance System

**Characteristic**	**Unweighted, No.^a^**	**Overall Weighted, % (95% CI)**	**Adults With Teeth Removed Due to Decay or Gum Disease,Weighted % (95% CI)**		

			**0 Teeth**	**1 to 5 Teeth**	**≥6 Teeth**
**Have diabetes**					
Yes	7,983	4.6 (4.4-4.8)	2.9 (2.7-3.1)	6.3 (5.9-6.7)	11.6 (10.6-12.7)
No	147,297	95.4 (95.2-95.6)	97.1 (96.9-97.3)	93.7 (93.3-94.1)	88.4 (87.3-89.4)
**No. teeth removed **					
0	90,943	61.8 (61.3-62.2)	NA	NA	NA
1 to 5	48,715	29.8 (29.3-30.2)	NA	NA	NA
6 or more	15,622	8.5 (8.2-8.7)	NA	NA	NA
**Age group, y**					
18 to 44	69,145	55.7 (55.2-56.2)	67.8 (67.2-68.3)	41.9 (41.0-42.7)	16.3 (15.0-17.5)
45 to 64	58,478	31.3 (30.9-31.8)	25.5 (25.0-26.0)	39.8 (39.0-40.7)	44.1 (42.6-45.6)
≥65	26,686	13.0 (12.7-13.2)	6.7 (6.5-7.0)	18.3 (17.7-18.9)	39.7 (38.2-41.1)
**Sex**					
Male	59,487	47.9 (47.4-48.4)	48.1 (47.5-48.8)	48.3 (47.4-49.2)	45.0 (43.5-46.5)
Female	95,793	52.1 (51.6-52.6)	51.9 (51.2-52.5)	51.7 (50.8-52.6)	55.0 (53.5-56.5)
**Race**					
Black	11,460	9.5 (9.2-9.8)	7.4 (7.0-7.8)	12.5 (11.9-13.1)	14.0 (12.8-15.1)
Other	8,960	8.4 (8.0-8.7)	8.2 (7.8-8.7)	9.2 (8.5-9.8)	6.8 (5.8-7.9)
White	133,644	82.1 (81.7-82.6)	84.4 (83.9-84.9)	78.3 (77.5-79.1)	79.2 (77.8-80.6)
**Annual income, $**					
<15,000	7,797	6.6 (6.3-6.9)	6.0 (5.5-6.4)	6.6 (6.1-7.2)	11.2 (10.0-12.5)
15,000 to <25,000	17,932	12.7 (12.4-13.1)	10.3 (9.9-10.8)	15.4 (14.7-16.2)	21.0 (19.6-22.4)
25,000 to <35,000	18,212	12.1 (11.7-12.4)	10.6 (10.2-11.0)	13.8 (13.2-14.5)	16.8 (15.6-17.9)
≥35,000	92,589	68.6 (68.1-69.1)	73.1 (72.5-73.8)	64.1 (63.2-65.0)	51.0 (49.3-52.6)
**Education**					
Less than high school	8,493	7.3 (7.0-7.6)	5.6 (5.2-6.0)	9.2 (8.6-9.8)	13.4 (12.1-14.6)
High school graduate	41,935	27.0 (26.6-27.4)	22.9 (22.3-23.5)	32.1 (31.3-32.9)	39.3 (37.8-40.7)
Some college	41,960	26.9 (26.4-27.3)	26.9 (26.3-27.5)	27.4 (26.6-28.2)	24.7 (23.5-25.9)
College graduate	62,669	38.8 (38.3-39.3)	44.6 (44.0-45.2)	31.3 (30.5-32.1)	22.7 (21.4-24.0)
**Employed or self-employed**					
Yes	105,382	68.9 (68.4-69.3)	72.5 (71.9-73.1)	66.9 (66.1-67.7)	49.2 (47.7-50.8)
No	49,596	31.1 (30.7-31.6)	27.5 (26.9-28.1)	33.1 (32.3-33.9)	50.8 (49.2-52.3)
**Married**					
Yes	94,267	63.1 (62.7-63.6)	61.6 (61.0-62.3)	66.3 (65.5-67.1)	63.4 (61.9-64.8)
No	60,515	36.9 (36.4-37.3)	38.4 (37.7-39.0)	33.7 (32.9-34.5)	36.6 (35.2-38.1)
**Smoked at least 100 cigarettes in lifetime**					
Yes	63,959	39.6 (39.1-40.0)	33.4 (32.8-33.9)	46.6 (45.8-47.5)	60.2 (58.7-61.7)
No	90,931	60.4 (60.0-60.9)	66.6 (66.1-67.2)	53.4 (52.5-54.2)	39.8 (38.3-41.3)
**Cost prevented access to doctor within past year**					
Yes	11,804	8.1 (7.9-8.4)	7.0 (6.7-7.4)	9.9 (9.4-10.5)	10.1 (9.1-11.1)
No	143,316	91.9 (91.6-92.1)	93.0 (92.6-93.3)	90.1 (89.5-90.6)	89.9 (88.9-90.9)
**Can identify at least one primary health care provider**					
Yes	134,452	83.7 (83.3-84.1)	82.7 (82.1-83.3)	84.6 (83.9-85.3)	88.0 (86.8-89.2)
No	20,559	16.3 (15.9-16.7)	17.3 (16.7-17.9)	15.4 (14.7-16.1)	12.0 (10.8-13.2)
**Have health care coverage**					
Yes	141,357	89.2 (88.8-89.5)	90.0 (89.5-90.5)	87.5 (86.9-88.2)	88.9 (87.7-90.1)
No	13,575	10.8 (10.5-11.2)	10.0 (9.5-10.5)	12.5 (11.8-13.1)	11.1 (9.9-12.3)

CI indicates confidence interval; NA, not applicable.

a Not all categories add to 155,280 because data on respondents who refused to answer a question or those who answered "Don't know" were not included.

**Table 2:** Diabetes and Tooth Removal Due to Decay or Gum Disease, by Number of Teeth Removed, Among Respondents Who Reported a Dental Visit in the Past Year and No Activity Limitation, 2004 Behavioral Risk Factor Surveillance System

**Characteristic**	OR (95% CI)	

	1 to 5 Teeth vs 0 Teeth Removed	≥6 Teeth (But Not All) vs 0 Teeth Removed
**Crude model**		
Have diabetes	1.64 (1.49-1.80)	3.16 (2.82-3.55)
Do not have diabetes	Ref	Ref
**Adjusted model^a^**		
Have diabetes	1.11 (0.99-1.24)^b^	1.46 (1.27-1.68)^c^
Do not have diabetes	Ref	Ref
**Stratified model for people with diabetes vs people without diabetes, by age group** d		
18 to 44 y	1.46 (1.15-1.86)	3.21 (2.05-5.00)
45 to 64 y	1.13 (0.97-1.31)	1.41 (1.15-1.73)
≥65 y	0.95 (0.79-1.13)	1.19 (0.99-1.44)

OR indicates odds ratio; CI, confidence interval; Ref, referent group.

a Adjusted for age group, race, sex, income, education, employment, marital status, cost barriers, ability to identify primary health care provider, health care coverage, and having smoked at least 100 cigarettes in lifetime.

b C statistic = 0.65.

c C statistic = 0.81.

d Adjusted for race, sex, income, education, employment, marital status, cost barriers, ability to identify primary health care provider, health care coverage, and having smoked at least 100 cigarettes in lifetime.
